# Multi-cohort metagenomics reveals strain functional heterogeneity and demonstrates fecal microbial load correction improves colorectal cancer diagnostic models

**DOI:** 10.3389/fmicb.2025.1656016

**Published:** 2025-09-24

**Authors:** Qiucheng Li, Fang Liu, Jianfeng Zhong, Xiaoling Fang, Xinyi Zhang, Huizhen Xiong, Guangyi Li, Honglei Chen

**Affiliations:** ^1^Digestive Endoscopy Center, The Eighth Affiliated Hospital, Sun Yat-sen University, Shenzhen, China; ^2^College of Biology, Hunan University, Changsha, China

**Keywords:** colorectal cancer, gut microbiome, strain, metagenomics, fecal microbial load, classification models

## Abstract

**Introduction:**

Colorectal cancer (CRC) is strongly associated with alterations in the gut microbiome. While numerous studies have examined this association, most focus on genus– or species–level taxonomic classifications, overlooking functional heterogeneity at the strain level.

**Methods:**

We integrated 1,123 metagenomic samples from seven global CRC cohorts to conduct multi-level metagenome-wide association studies (MWAS). Fecal microbial load (FML) correction was applied to mitigate technical confounding. We evaluated the performance of taxonomic models at various resolutions strain, species, and genus levels in classifying CRC status both within and across cohorts.

**Results:**

Strain–level analysis revealed conspecific strains with divergent associations to CRC. For instance, distinct strains of Bacteroides thetaiotaomicron exhibited both protective and risk-increasing effects across different cohorts. Genomic functional annotation suggested potential mechanistic bases for these opposing roles. Correction for FML reduced confounding and significantly improved the performance of within–cohort and cross–cohort CRC classification models. Interestingly, genus- and species-level models demonstrated superior predictive robustness compared to strain–level models, likely due to higher microbial abundance and greater cross-population conservation at these taxonomic ranks.

**Conclusion:**

Our study underscores the biological relevance of strain level analysis in elucidating functional diversity within the microbiome. However, higher taxonomic levels provide more robust and clinically translatable diagnostic markers for CRC. Integrating FML correction with multi-level taxonomic profiling enhances both mechanistic insight into microbiom CRC interactions and the generalizability of diagnostic models across diverse populations.

## Introduction

Colorectal cancer (CRC), comprising approximately 10% of all cancer cases worldwide, stands as the second leading cause of cancer-related mortality (Sung et al., 2021). Due to its typically asymptomatic early stages, CRC is often diagnosed at advanced phases when therapeutic options are limited. Accumulating evidence over recent years has firmly established a critical association between CRC development and the human gut microbiome, with interactions primarily mediated through mechanisms such as microbial metabolism, inflammatory regulation, immune dysbalance, and intestinal barrier dysfunction (White and Sears, 2024; Wong and Yu, 2023). Studies have further highlighted the potential of gut microbes as diagnostic biomarkers, demonstrating high accuracy in predicting gastrointestinal diseases including CRC and inflammatory bowel disease (IBD) (Wong et al., 2017; Wang and Jia, 2016).

Notably, most existing research has focused on taxonomic analysis at the genus or species level, overlooking the functional heterogeneity that may exist among different strains within the same genus or species. Strains from the same microbial species can exhibit divergent phenotypes or even opposing biological functions in host environments. For example, *Escherichia coli* (*E. coli*), a common commensal in mammalian intestines, includes the probiotic strain Nissle 1917, which synthesizes essential vitamins, alongside highly pathogenic variants like *E. coli* STEC O26:H11 and EHEC O104:H4, associated with hemolytic uremic syndrome and fatal diarrhea (Marx, 2016; Bonanno et al., 2015). Similarly, distinct strains of *Staphylococcus aureus* and *Streptococcus pyogenes* elicit markedly different immune responses in humans (Sela et al., 2018; Van Rossum et al., 2020).

Advances in metagenomic sequencing depth and high-resolution taxonomic profiling tools have enabled strain-level metagenome-wide association studies (MWAS), providing new avenues to dissect microbiome functional characteristics (Shi et al., 2022; Olm et al., 2021). However, a gap remains in strain-resolved MWAS of CRC, particularly regarding systematic cross-cohort comparisons across diverse geographical populations.

Additionally, fecal microbial load (FML), an important factor influencing microbial composition analysis, has gained increasing attention. A recent study demonstrated that neglecting FML correction can lead to spurious associations between microbial taxa and diseases, with effect sizes and significance metrics changing substantially after load adjustment (Nishijima et al., 2025). This suggests FML represents a potential confounder in MWAS, yet its impact on disease classification model performance—especially across taxonomic levels (strain, species, and genus) and geographical cohorts—remains uncharacterized.

To address these knowledge gaps, our study integrated 1,123 samples from seven independent CRC cohorts across seven countries, employing a standardized analytical pipeline to conduct systematic MWAS at strain, species, and genus levels. We evaluated the effects of FML correction on the identification of disease-associated microbial features and the performance of classification models for CRC. Through multi-cohort, multi-level comparisons, we aimed to: (1) demonstrate the unique value of strain-level analysis in resolving biological heterogeneity, (2) assess the robustness of genus/species-level features in diagnostic models for clinical utility, and (3) determine whether FML correction enhances disease prediction performance. Our findings provide critical insights for mechanistic studies of the gut microbiome in CRC and its application in early clinical diagnosis.

## Materials and methods

### Cohort selection

We collected published fecal whole metagenome sequencing (WMS) data from seven cohorts consisting of CRC patients and healthy controls, spanning seven countries. Raw sequencing data for these samples were downloaded from the Sequence Read Archive (SRA) and European Nucleotide Archive (ENA) using the following accession IDs: ERP008729 (AUT cohort) from Feng et al. (2015), PRJEB10878. (CHI cohort) from Yu et al. (2017), PRJNA531273 and PRJNA397112 (IND cohort) from Gupta et al. (2019), ERP005534 (FRA cohort) from Zeller et al. (2014), SRP136711 (ITA cohort) from Thomas et al. (2019), PRJEB12449 (USA cohort) from Vogtmann et al. (2016), and DRA006684/DRA008156 (JPN cohort) from Yachida et al. (2019). Metadata were manually curated from original studies, excluding samples with missing Age, BMI, or Gender information, resulting in 1,123 samples. Only colorectal cancer and healthy control samples were included in downstream analyses, excluding adenoma cases.

### Sample preprocessing and metagenomic profiling

For raw sequencing data preprocessing, KneadData (https://github.com/biobakery/kneaddata, V0.12.0) was used for quality control and host contamination removal. Trimmomatic (V0.39, integrated in KneadData) performed sequence quality filtering and adapter trimming with parameters: ILLUMINACLIP:TruSeq3-PE.fa:2:40:15 SLIDINGWINDOW:4:20 MINLEN:50. Host-derived reads were removed by aligning to the human reference genome (GRCh38_p14) using Bowtie2 (V2.4.1) with parameters: –very-sensitive –dovetail –reorder.

Strain-level abundance analysis of preprocessed sequences was conducted using Sylph (V0.6.1) (Shaw and Yu, 2024) against a custom non-redundant strain database (c200_gtdb_strain.syldb, compression parameter c = 200). Genomes were downloaded from the Genome Taxonomy Database (GTDB) using genome_updater (https://github.com/pirovc/genome_updater), with a limit of 100 genomes per species to constrain computational costs, yielding 343,362 strains. For each species, pairwise average nucleotide identity (ANI) matrices were calculated by FastANI (v1.33) (Jain et al., 2018), followed by custom graph-based clustering at ANI thresholds of 95%–99.9%. Here, the 95% ANI threshold is widely used for microbial species delineation to differentiate interspecies boundaries (Jain et al., 2018; Konstantinidis and Tiedje, 2005; Goris et al., 2007), while the 99.9% upper bound is designed to capture intraspecies strain-level genetic variations, preventing the loss of biologically meaningful diversity due to overly stringent thresholds. The refined database contained 206,273 strains (GTDB:206273).

Species-level analysis utilized MetaPhlAn4 (V4.1.1) (Blanco-Ḿıguez et al., 2023) with the mpa_vJan21_ CHOCOPhlAnSGB_202103 reference database. Taxonomic results from both tools were merged at genus, species, and strain levels using MetaPhlAn's merge_metaphlan_tables.py script for downstream differential analysis.

### Fecal microbial load prediction

Fecal microbial load (total microbial cells per gram or cell density) was predicted using the Microbial Load Predictor (MLP, https://microbiome-tools.embl.de/mlp/) (Nishijima et al., 2025), an R-based computational tool designed to estimate FML from species-level taxonomic profiles of the human gut microbiome. Input files consisted of species-level taxonomic feature tables generated by classification tools including mOTUs v2.5, mOTUs v3.0, MetaPhlAn3, MetaPhlAn4, or RDP-based 16S rRNA annotations. Given the demonstrated congruence between species-level classifications from MetaPhlAn4 and Sylph, we utilized the metagenomic profiling outputs of MetaPhlAn4 to predict the fecal microbial load.

### Microbiome diversity and community structure analysis

Alpha-diversity metrics (Shannon index, Richness) were calculated using the vegan package (V2.6-8) (Oksanen, 2022). Multivariate linear regression models [lm() function] analyzed covariate effects on diversity, reporting coefficients, standard errors, and p-values with residual diagnostics. Group differences were tested via Wilcoxon rank-sum tests. Beta-diversity was assessed using Bray-Curtis distance-based permutational multivariate analysis of variance (PERMANOVA) (Anderson, 2014) via adonis2 in vegan, evaluating independent contributions of covariates (Disease, Age, Gender, BMI, and FML) with 999 permutations. Pairwise comparisons of significant variables (e.g., Disease) used pairwise.adonis() from the pairwiseAdonis package, with p-values corrected for false discovery rate (FDR) via Benjamini–Hochberg. These analyses were conducted using R scripts.

### Training-test set partitioning

Samples from each country were partitioned into training and test sets at an 8:2 ratio, with the random grouping process repeated 100 times to construct diverse datasets. Stratified sampling was employed in each partition to ensure balanced class representation in the test set, maintaining proportional distribution of colorectal cancer (CRC) and non-CRC cases. To preserve consistency between the feature matrix (microbial abundance data) and metadata, microbial abundance matrices were extracted according to the partition results. All partitioned datasets (including metadata and microbial profiles) were stored in a predefined directory structure for subsequent model training and validation.

### Differential abundance analysis

Multivariate Association with Linear Models 2 (MaAsLin2, V1.20.0) (Mallick et al., 2020) was used to identify microbial features associated with CRC status. We utilized OTU abundance tables generated by Sylph and MetaPhlAn4, combined with sample metadata including disease status, age, gender, BMI, and total fecal microbial load (FML). Two model types were constructed: one excluding FML as a covariate and another including it to assess its regulatory effect. Linear regression models were applied with log-transformed feature data, using raw relative abundances (where the sum of relative abundances for each taxonomic level OTU in a single sample equals 1) without normalization. Features were filtered to require a minimum occurrence frequency of 10%, and multiple hypothesis testing was corrected using the Benjamini-Hochberg method with a significance threshold FDR < 0.25.

### Functional annotation of strain genomes

Functional annotation of bacterial genomes was performed using three databases: VFDB (Zhou et al., 2025), CARD (Alcock et al., 2023), and KEGG (Kanehisa and Goto, 2000). For VFDB, Abricate (V1.0.1) (Seemann, 2017) was applied to genome FASTA files to identify virulence factors, with a minimum sequence identity and coverage set to 50%. For CARD, Resistance Gene Identifier (RGI, V6.0.4) (Alcock et al., 2023) was used with the CARD database to predict antibiotic resistance genes, using contig input and DIAMOND for sequence alignment. For KEGG, gene prediction and general functional annotation were performed with Prokka (V1.14.6) (Seemann, 2014), followed by functional annotation of predicted protein sequences and KEGG pathway assignment using EGGNOG-mapper (V2.0.1) (Cantalapiedra et al., 2021). Differential pathway enrichment was evaluated by Fisher's exact test with FDR-adjusted *p*-values.

### Batch effect correction

Microbial abundance data were first filtered to remove low-abundance OTUs (retaining only OTUs present in at least 10% of samples at the genus and species levels; for strains, due to low abundance and high specificity, OTUs present in at least 1% of samples were retained) and then matched with sample metadata. To correct for cohort-associated batch effects while preserving disease-related signals, compositional data were subjected to centered log-ratio (CLR) transformation, which is suitable for handling the compositionality, sparsity, and skewness of microbiome data. Batch effects were subsequently adjusted using the ComBat (implemented in the sva R package, V3.54.0) (Johnson et al., 2007) method, with cohort as the batch variable and disease status as a covariate. The effectiveness of batch correction was assessed using PERMANOVA, and PCA was applied to visualize the data before and after correction. The batch-corrected relative abundance data were then used for downstream analyses.

### Disease classifier construction and validation

Random Forest (RF) (Breiman, 2001) models were employed in conjunction with Recursive Feature Elimination with Cross-Validation (RFECV) and hyperparameter optimization to evaluate the classification performance of microbiome features in predicting colorectal cancer (CRC). Two independent modeling approaches were implemented based on feature subsets derived from differential abundance analysis (with and without fecal microbial load correction). For each modeling approach, RFECV was first applied to the training set to identify stable discriminatory features. Hyperparameters of the RF models were then optimized via randomized search (RandomizedSearchCV() function in Python package of sklearn) over a predefined parameter grid. Model performance was evaluated on the test set, and confidence intervals for the Area Under the Receiver Operating Characteristic Curve (AUC) were estimated using bootstrap resampling. Pairwise comparisons of AUC distributions were conducted using the non-parametric Mann-Whitney U test: (i) between models incorporating vs. excluding total microbial load adjustment, and (ii) across taxonomic levels (genus, species, and strain). Statistical significance cutoff was set as *P* ≤ 0.05. Cross-cohort external validation employed a leave-one-country-out strategy, where models were trained on data from one country and independently validated on each of the remaining six countries. This approach ensured that each national cohort served sequentially as the training set, with the other six cohorts functioning as distinct validation sets to assess model generalizability across geographical populations. Only features retained during training were used in validation; missing features were imputed with zeros to maintain dimensional consistency. If all required features were absent in a validation set, the corresponding model-validation combination was excluded.

## Results

### Cohort characteristics and multilevel microbiota diversity analysis

**Figure 1:**
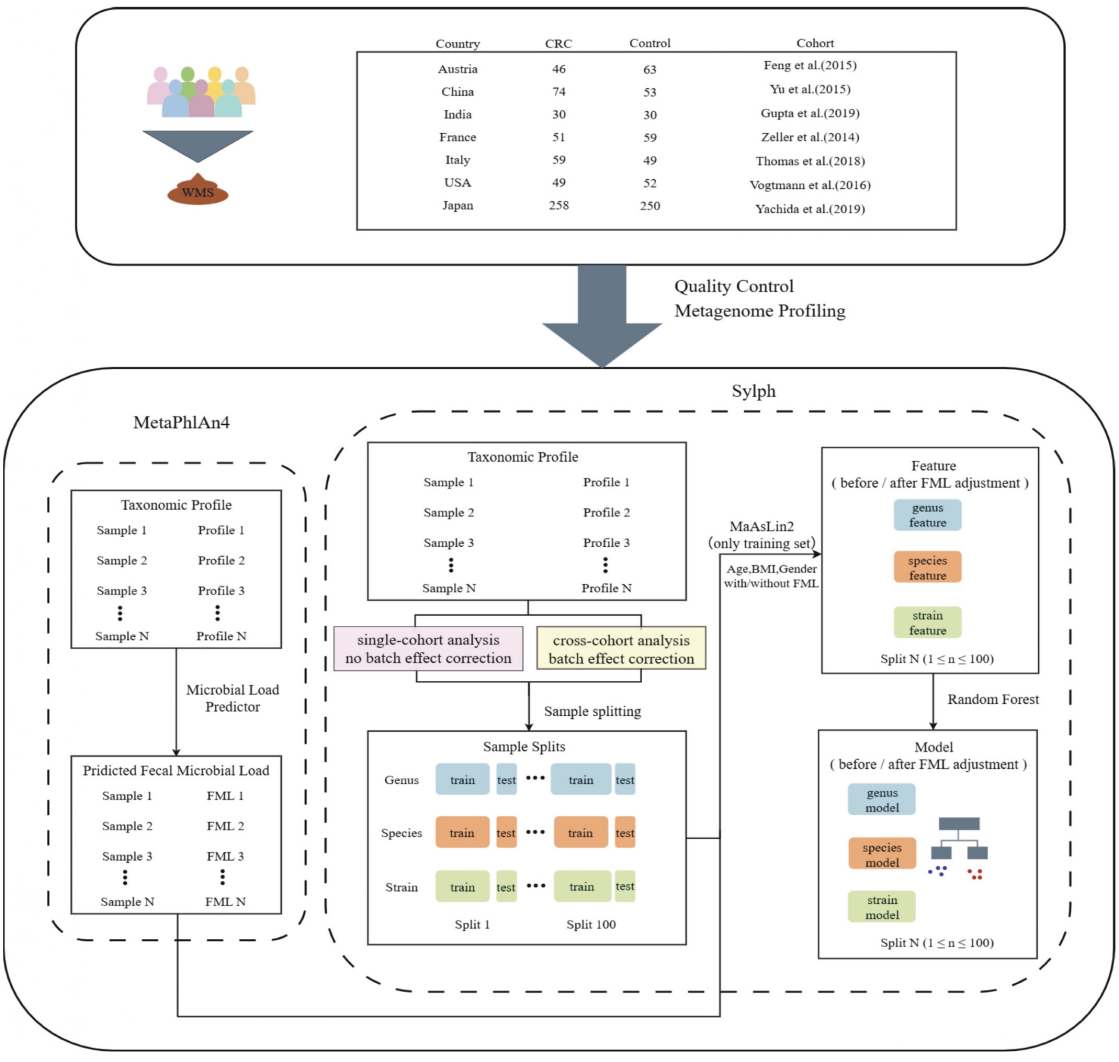
Workflow diagram of this study. Fecal samples from colorectal cancer (CRC) patients and healthy controls were collected from seven countries and subjected to whole-metagenome shotgun sequencing (WMS). After quality control of sequencing files, taxonomic profiling and microbial load estimation were performed using MetaPhlAn4. In parallel, Sylph was used to annotate microbial features at the genus, species, and strain levels, generating abundance matrices at each taxonomic level: no batch effect correction was applied for single-cohort analysis, whereas batch effect correction was performed for cross-cohort analysis. Samples were randomly split into training and test sets and repeated 100 times at each taxonomic level. For each training set, MaAsLin2 was used to adjust for confounding factors including age, BMI, gender, and fecal microbial load (FML) to identify associated microbial markers. Features were categorized into *before FML adjustment* (unadjusted) and *after FML adjustment* (adjusted) based on fecal microbial load correction. Finally, random forest models were constructed based on the features for both single-cohort and cross-cohort analyses to classify CRC and compare model performance.

Raw metagenomic sequencing data from all 1,123 samples across seven independent cohorts were first processed using a uniform standardized pipeline to ensure comparability, involving quality control, adapter trimming, and metagenomic profiling (detailed in Methods, see Figure 1 and Supplementary Table 1). Strain-level analysis leveraged the metagenomic classifier Sylph to construct a non-redundant reference genome database from the Genome Taxonomy Database (GTDB), while genus- and species-level taxonomic annotations were performed using MetaPhlAn4 and Sylph, respectively. Fecal microbial load (FML) was estimated via the Microbial Load Predictor pipeline (MLP, https://microbiome-tools.embl.de/mlp/) (Nishijima et al., 2025), which requires input in mOTU or MetaPhlAn format; given the demonstrated consistency between Sylph and MetaPhlAn4 at the species annotation level, we utilized MetaPhlAn4 outputs for FML estimation and included load as a covariate in subsequent statistical models.

In the subsequent experiments, to mitigate biases from random dataset partitioning, each cohort was stratified and randomly divided into training (80%) and test (20%) sets using an 8:2 ratio, with this process repeated 100 times. Training data were used for identifying differential microbial features via MaAsLin2 and constructing random forest classifiers, while test sets enabled within-cohort validation of model performance.

**Figure 2:**
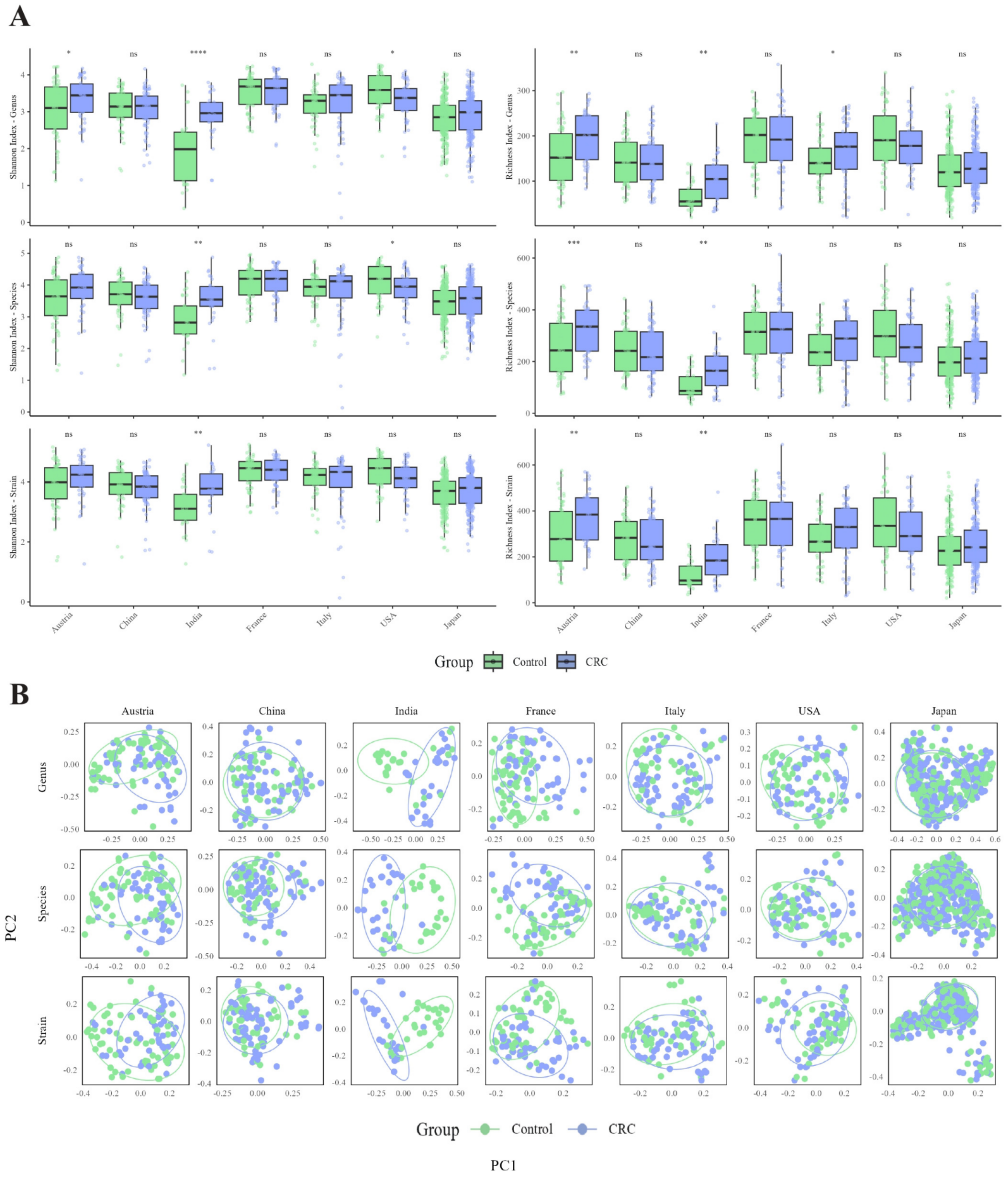
**(A)** Comparison of alpha diversity indices (Shannon and Richness) between CRC and control groups across different taxonomic levels (genus, species, strain) in each country. The Shannon index reflects both the richness and evenness of taxa, while the Richness index represents the number of unique taxa. Statistical significance was assessed using the non-parametric Wilcoxon rank-sum test. Significance levels: ^****^p < 0.0001; ^***^p < 0.001; ^**^p < 0.01; ^*^p < 0.05; ns, not significant. **(B)** Principal component analysis (PCA) of genus-, species-, and strain-level microbial profiles in CRC and control samples from different countries. Each subplot shows the distribution of samples along the first two principal components (PC1 and PC2) with 95% confidence ellipses for each group.

Analysis of within-sample diversity (α-diversity) revealed heterogeneous patterns across geographical cohorts (Figure 2A). In Indian and Austrian samples, CRC cases exhibited significantly higher Shannon diversity and richness indices than controls at the genus, species, and strain levels (*P* < 0.05 for all comparisons), whereas most other cohorts showed minimal or non-significant differences in microbial diversity between groups. These findings highlight the geographical dependency and complexity of CRC-associated gut microbiota alterations. Linear regression models further showed that FML exerted a significant effect on both Shannon and richness metrics across all taxonomic levels in all cohorts except India (*P* < 0.05, Supplementary Tables 2, 3), underscoring the need to account for microbial load when interpreting diversity indices.

As for β-diversity analysis, principal coordinate analysis (PCoA) based on Bray-Curtis dissimilarity mirrored α-diversity trends, with notable distinctions in microbial community structure between CRC cases and controls (Figure 2B). Significant β-diversity differences (permutation test, *P* < 0.05) were observed at all taxonomic levels in most cohorts, except for Italian and US samples, indicating region- or population-specific shifts in gut microbiota associated with CRC. When FML was incorporated as a covariate in permutational multivariate analysis of variance (PERMANOVA), it emerged as a significant factor influencing community structure across all taxonomic levels and cohorts (*P* < 0.05, Supplementary Tables 4, 5), confirming its role as a critical confounder in microbiome compositional analyses.

### Contrasting effects of conspecific strains in colorectal cancer

**Figure 3:**
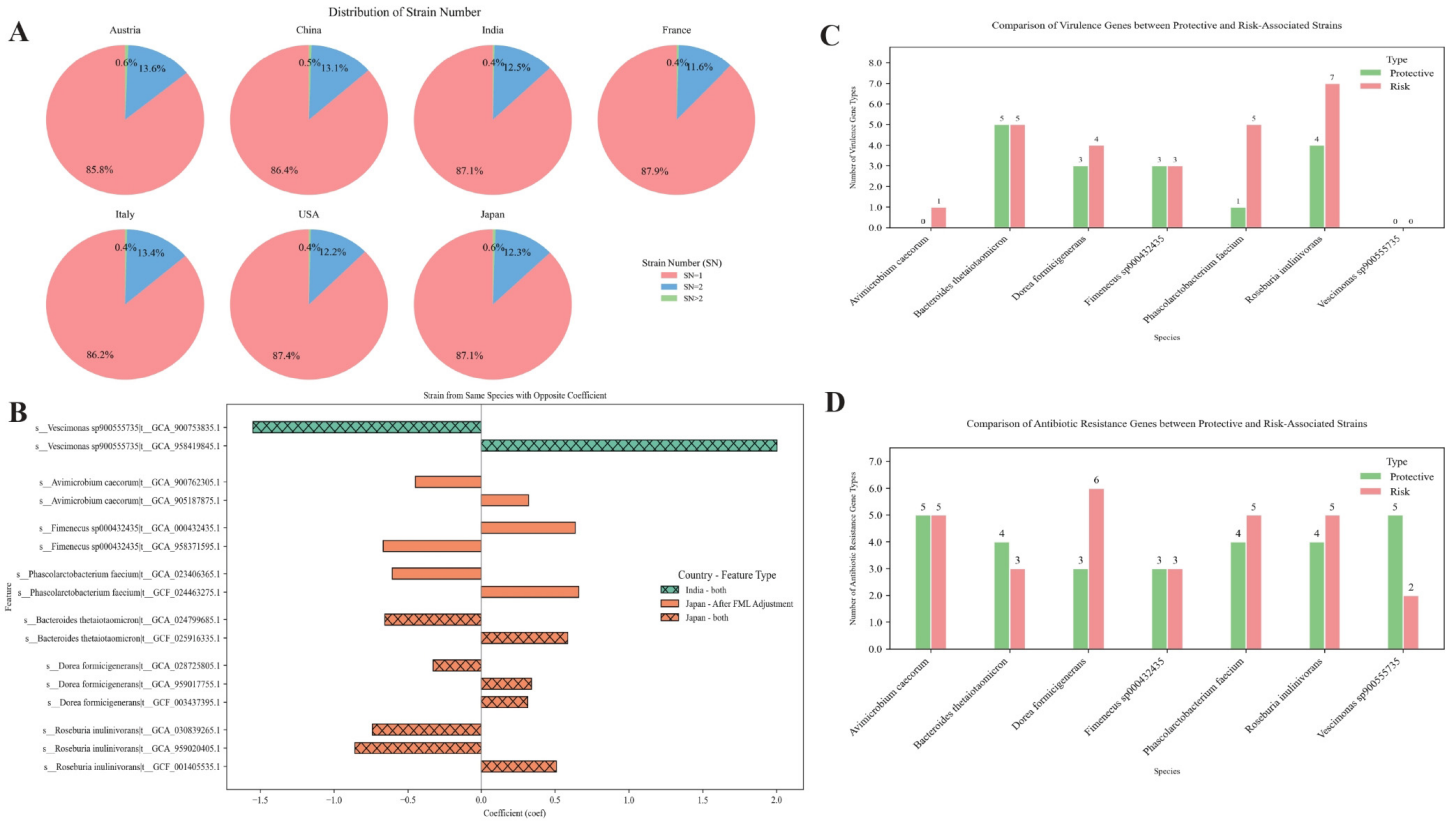
**(A)** Distribution of strain richness for each species across all samples from the seven countries. Strain Number (SN) indicates the number of strains identified within the same species. **(B)** Opposite correlation coefficients with CRC among strains from the same species across different cohorts, identified by MaAsLin2 analysis. Positive values indicate a positive association with CRC, while negative values indicate a negative association. False discovery rate (FDR) < 0.25 was used as the threshold for statistical significance. **(C)** Comparison of virulence gene types between CRC-associated risk strains and protective strains within the same species. **(D)** Comparison of antibiotic resistance gene types between CRC-associated risk strains and protective strains within the same species.

Previous studies have referred to this metric as strain richness (SR), defined as the number of strains of a given microbial species *j* present in the gut of an individual *i*, denoted as SR_*ij*_. Typically, an individual harbors no more than two strains per species. To distinguish this from the “richness” measure used in Result 1, we hereafter denote this concept as strain number (SN) (Chen-Liaw et al., 2025). In our analysis, the proportion of samples with SN ≥2 across all cohorts was consistently below 15%, and 91% of 1,123 total samples exhibited fewer than 20% species with SN ≥2. The majority of SN ≥2 cases (overwhelmingly SN = 2) aligned with historical observations (Figure 3A, Supplementary Table 6). Using MaAsLin2, we identified strain-level associations with CRC across cohorts and observed striking functional dichotomy within species. In all cohorts except the USA, two or more strains belonging to the same species were detected. Notably, in the Indian and Japanese cohorts, certain species such as *Vescimonas* sp900555735, *Avimicrobium caecorum, Bacteroides thetaiotaomicron*, and *Dorea formicigenerans* exhibited opposing strain-level associations with CRC risk, as indicated by their regression coefficients (Figure 3B, Supplementary Table 7).

To further explore the biological mechanisms underlying opposing effects on CRC among strains from the same species, we performed functional annotation of their genomes using VFDB (Virulence Factor Database) (Zhou et al., 2025), CARD (Comprehensive Antibiotic Resistance Database) (Alcock et al., 2023), and KEGG (metabolic pathway database) (Kanehisa and Goto, 2000). We compared “risk strains” and “protective strains” in terms of metabolic pathways and virulence gene content.

Based on presence/absence data of genes as annotated in the VFDB and CARD (Supplementary Tables 8, 9) , the results show that risk strains generally carry a more diverse set of virulence factor genes (VFGs) and antibiotic resistance genes (ARGs) (Figures 3C, D). Specifically, VFDB data showed that risk strains carry VFGs including fliP and rfaD. fliP is involved in flagellar protein transport and assembly and may induce chronic inflammation via the TLR5/NF-κB pathway (Song et al., 2017). *rfaD* is involved in the biosynthesis of bacterial lipopolysaccharide (LPS). LPS can activate the host immune system and trigger inflammatory responses; under chronic inflammatory conditions, LPS may continuously stimulate the intestinal immune system, thereby leading to persistent inflammation (Zhang et al., 2024). Other VFGs, such as cps4I and pseB, may promote bacterial colonization, immune evasion, and host inflammatory responses. Although these genes are not classical oncogenes, their presence in the gut microbiota or specific risk strains may indicate a microenvironment conducive to low-grade chronic inflammation, which is a known risk factor for CRC (Shimomura et al., 2023). CARD data showed that ARGs such as ACI-1, dfrF, and tet(O/W/40) may reflect community structures associated with dysbiosis and increased microbial activity.

In the KEGG pathway analysis, we compared the enrichment of pathways between risk strains and protective strains (Supplementary Table 10). Considering the high ANI similarity among strains of the same species, the FDR threshold was relaxed to 0.25 to avoid missing potentially relevant differences. The results showed that map00540, which is related to lipopolysaccharide biosynthesis, may induce chronic inflammation via the TLR4/NF-κB signaling pathway (Hu et al., 2021; Luo and Zhang, 2017), thereby increasing CRC risk, and map05111, which is related to Vibrio cholerae infection, may contribute to carcinogenesis by sustaining chronic infection and local inflammation (Ou et al., 2009). By contrast, map00511, which is related to other glycan degradation, may help maintain normal glycosylation levels and reduce abnormal glycosylation-associated changes in cell adhesion and metastasis (Bangarh et al., 2023), whereas map00600, which is related to sphingolipid metabolism, may modulate the ceramide–S1P balance (Karmelić et al., 2024), promoting apoptosis and exerting anti-inflammatory effects.

These findings highlight the utility of strain-level analysis in resolving microbiome functional heterogeneity, which is obscured at the species level. The identification of conspecific strains with diametrically opposed effects on CRC provides a rational basis for developing strain-targeted therapeutic interventions. Although we proposed hypotheses for some key pathways and genes, other pathways and genes not detailed here may also be involved in microbiota-mediated inflammation and carcinogenesis, and future studies are needed to clarify their specific roles and mechanisms.

### FML correction improves performance of disease classifiers

**Figure 4:**
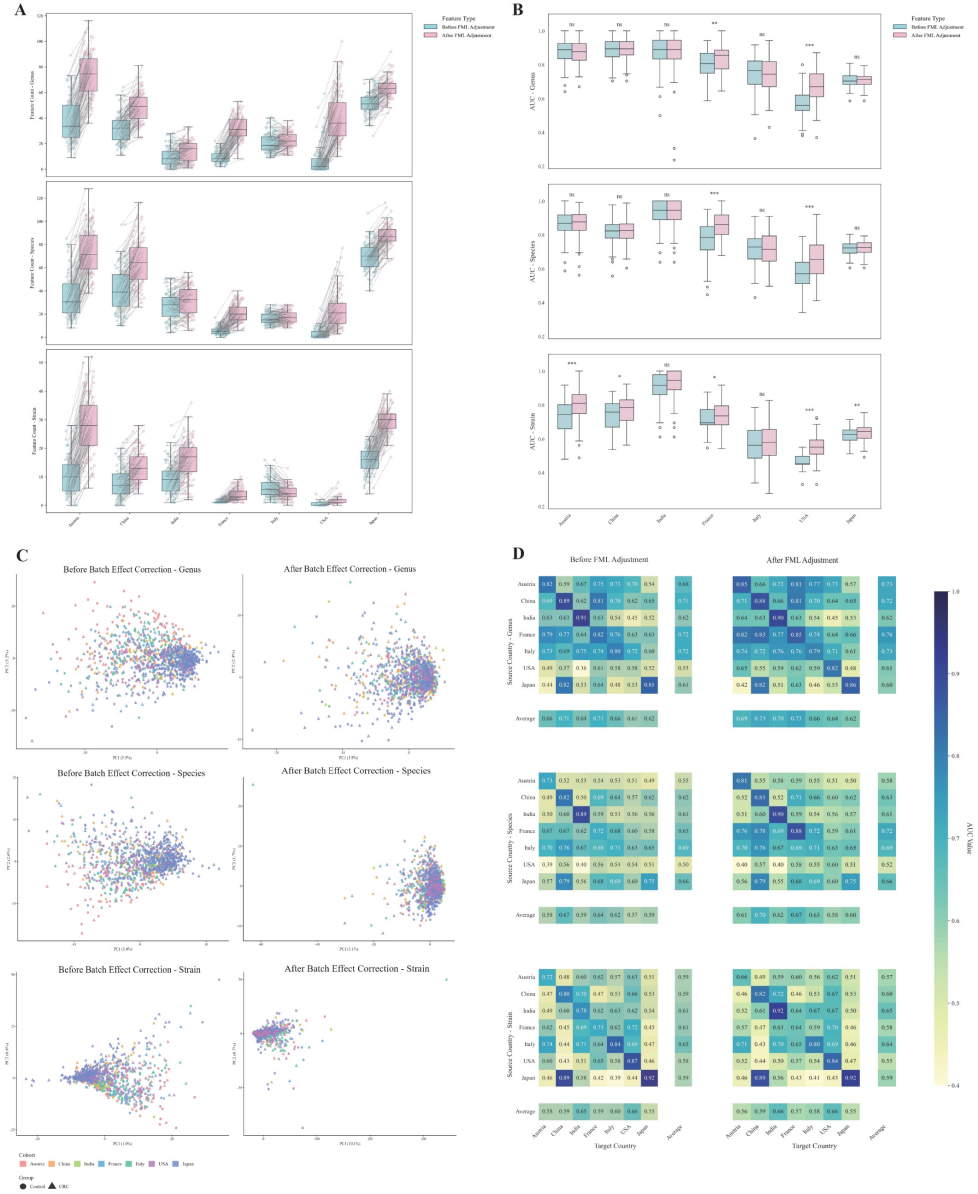
**(A)** Number of differential features between CRC and control groups identified by MaAsLin2 at the genus, species, and strain levels before and after microbial load adjustment across countries. **(B)** Comparison of classification performance (area under the ROC curve, AUC) of CRC prediction models before and after microbial load adjustment across countries. Significance levels: ^***^p < 0.001; ^**^p < 0.01; ^*^p < 0.05; ns, not significant. Statistical significance was assessed using the non-parametric Mann–Whitney U test. **(C)** PCA of the samples after batch effect correction. Each point represents a sample, and colors correspond to different study cohorts. **(D)** Cross-cohort validation performance of CRC classification models before and after microbial load adjustment.

Significant features selected by MaAsLin2 at the strain, species, and genus levels from the training set were input into classification models, and the test sets were used for within-cohort validation of model performance. Across cohorts, fecal microbial load (FML) correction increased the number of detected differential features in all groups except Italy, with genus- and species-level features consistently outnumbering strain-level equivalents (Figure 4A, Supplementary Table 11). To address feature count variability, recursive feature elimination with cross-validation (RFECV) was applied to select subsets optimizing area under the receiver operating characteristic curve (AUC).

We systematically assessed model performance using multiple metrics, including AUC, accuracy, sensitivity, specificity, positive predictive value (PPV), negative predictive value (NPV), and F1 score. At the strain level, models constructed from FML-corrected features consistently outperformed their uncorrected counterparts across all cohorts, although the degree of statistical significance varied (Figure 4B, Supplementary Table 12). Similar trends were observed at the genus and species levels, where FML normalization consistently enhanced discriminative accuracy.

To further validate the reliability of conclusions derived from single-cohort models, we additionally performed cross-cohort validation. Batch effects were first corrected, and their influence on disease status at the genus, species, and strain levels was reduced from 0.123, 0.073, 0.058 to 0.016, 0.017, 0.010, respectively (Supplementary Table 13). We then applied principal component analysis (PCA) to assess clustering patterns before and after correction, and observed a markedly increased overlap across cohorts after correction, indicating that batch effects had been effectively controlled (Figure 4C). Based on the corrected data, we re-performed differential feature analysis and classifier construction, with cross-cohort results further corroborating the robustness of our initial conclusions (Figure 4D, Supplementary Table 14).

### Higher taxonomic levels outperform strain-level disease classifiers

**Figure 5:**
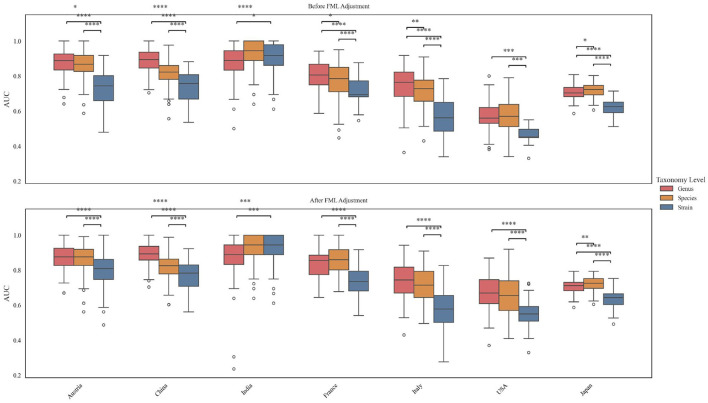
Comparison of AUC values of CRC classification models constructed at different taxonomic levels (strain, species, and genus). Statistical significance was determined using the Mann–Whitney U test. Significance levels: ***p < 0.001; **p < 0.01; *p < 0.05; ns, not significant.

We evaluated the impact of taxonomic resolution on classifier performance by comparing models built from genus-, species-, and strain-level features across multiple metrics. In all cohorts except the FML-corrected Indian dataset, genus-, and species-level models showed significantly better performance in AUC compared to strain-level models, regardless of microbial load adjustment (Figure 5). This trend was also observed in cross-cohort validation, where genus- and species-level models demonstrated greater generalizability across different geographic populations. All complete evaluation metrics, including accuracy, sensitivity, specificity, PPV, NPV, and F1 score, are provided in the shared Supplementary Table 12.

Two putative mechanisms may explain this observation: (1) Strain-level features typically exhibit lower relative abundances than higher taxonomic levels, leading to increased technical noise and reduced signal-to-noise ratios during metagenomic profiling; and (2) Strain-specific markers are highly influenced by individual host backgrounds (e.g., genetics, lifestyle) and geographical factors, limiting their transferability across diverse cohorts (Andreu-Sánchez et al., 2025). These findings highlight that while strain-level analysis uncovers biological heterogeneity, genus-, and species-level features offer more robust and reproducible signals for clinical diagnostic applications, balancing mechanistic insight with practical utility in multi-cohort settings.

## Discussion

This study leveraged 1,123 metagenomic samples from seven independent cohorts to systematically evaluate gut microbiome associations with colorectal cancer (CRC) across taxonomic levels (genus, species, and strain). We also investigated the impact of fecal microbial load (FML) correction on disease classification model performance and compared predictive capabilities across taxonomic resolutions.

Most cohorts showed higher Shannon diversity and richness indices in CRC patients compared to healthy controls, aligning with prior studies suggesting increased microbiota diversity in CRC (Figure 2A). However, this trend was not statistically significant in all cohorts, likely reflecting complex influences of population background, geographical environment, and study design on microbial community structure. Beta-diversity analysis revealed limited significant compositional differences between CRC cases and controls, implying that pronounced microbiome structural alterations may primarily occur in advanced disease stages (Figure 2B).

We observed that the majority of samples harbored no more than two strains per species (Figure 3A), and across multiple cohorts, distinct strains within the same species exhibited opposing associations with CRC risk (Figure 3B). This finding underscores strain-level functional heterogeneity, where conspecific strains can influence host health through divergent mechanisms. Strain-level analysis provides finer biological resolution than higher taxonomic levels, enabling identification of potentially pathogenic or protective strains. Furthermore, by functionally annotating the genomes of risk and protective strains within the same species, we proposed hypotheses regarding their potential biological mechanisms.

Fecal microbial load (FML) correction significantly improved both within-cohort and cross-cohort predictive performance of CRC classifiers (Figures 4B, D). Uncorrected models may be confounded by total microbial biomass, which distorts relative abundance measurements and masks true biological signals. Load adjustment mitigates this confound, allowing models to more accurately identify CRC-associated microbial features. These results advocate for routine inclusion of FML as a covariate in metagenomic analyses.

While strain-level analysis offers high biological resolution, genus- and species-level models outperformed strain-resolved counterparts in predictive accuracy (Figure 5). This discrepancy arises from: (1) lower strain-level abundances and associated technical noise, reducing signal-to-noise ratios; and (2) high inter-individual and geographical specificity of strains, limiting cross-cohort reproducibility. In contrast, genus/species-level features exhibit greater conservation across populations, making them more robust for clinical prediction. These findings highlight the need to balance research objectives when selecting taxonomic resolution: strain-level analysis for mechanistic insights, versus higher levels for stable diagnostic markers.

This work has several limitations: although we proposed hypotheses for some key pathways and genes, their specific roles and mechanisms still need to be further clarified; host genetic or clinical variables were not integrated into the models; and the findings are associative rather than causal. Future studies could leverage metagenome-assembled genomes (MAGs) and culturomics to characterize strain functions, and integrate multi-omics data to develop more comprehensive predictive models.

## Conclusion

Through multi-cohort integrative analysis, this study reveals taxon-level specificities in gut microbiome-CRC associations. Strain-level analysis uncovers functional heterogeneity invisible at higher taxonomic scales, but genus/species-level features currently offer greater stability for clinical translation. To further advance the field, future studies should leverage metagenome-assembled genomes (MAGs) and culturomics to better characterize strain functions, and integrate multi-omics data to develop more comprehensive and robust predictive models. Ultimately, improving high-sensitivity strain detection and functional validation methods will be critical for translating strain-resolved microbiome insights into precision medicine.

## Data Availability

Cohort metadata, analysis results, and code are available at Zenodo (https://doi.org/10.5281/zenodo.16899759) and GitHub (https://github.com/gygyli/CRC_analysis).
